# Protective Effects of Galla Rhois, the Excrescence Produced by the Sumac Aphid, *Schlechtendalia chinensis*, on Transient Focal Cerebral Ischemia in the Rat

**DOI:** 10.1673/031.012.0110

**Published:** 2012-01-27

**Authors:** Kyungjin Lee, Jinmo Kim, Beom-joon Lee, Jea-Woo Park, Kang-Hyun Leem, Youngmin Bu

**Affiliations:** ^1^ Department of Herbal Pharmacology, College of Oriental Medicine, Kyung Hee University, Seoul, 130–701, South Korea; ^2^ Kangnam Korean Hospital, Kyung Hee University, Seoul, 135–501, South Korea; ^3^ Internal Medicine, Kyung Hee Oriental Medical Hospital, Seoul, 130–701, South Korea; ^4^ Department of Herbal Pharmacology, College of Oriental Medicine, Semyung University, Jechon, 390–711, South Korea

**Keywords:** lipid-peroxidation, neuroprotective effects, radical scavenger, sensory motor deficits, transient focal cerebral artery occlusion

## Abstract

Galla Rhois is formed by aphids, primarily *Schlechtendalia chinensis* Bell (Homoptera: Pemphigidae), on the leaf of sumac, *Rhus javanica* L. (Sapindales: Anacardiaceae). It is a tannin-rich herb that is widely used in traditional Korean medicine. Its various pharmacological effects, including its radical-scavenging effects, have been reported. The purpose of the current study was to determine if these radical-scavenging effects can be confirmed using in vitro assays and to investigate its neuroprotective effects, optimal dosage, mechanisms, and therapeutic time window in an animal model of stroke. Galla Rhois 85% methanol extract (GRE) exhibited potent and dose-dependent radical-scavenging effects on various radicals. Oral administration of GRE (300 mg/kg) in a transient focal cerebral ischemia rat model (two hours of occlusion followed by 22 hours of reperfusion) reduced the brain infarct volume by 37.5%. It also improved sensory motor function and reduced lipid-peroxidation in middle cerebral artery occlusion. However, it did not have any inhibitory effects on brain edema. The time window study revealed that pre- and co-treatment with GRE had protective effects, but post-treatment with GRE (three or six hours after ischemia) did not have protective effects. In conclusion, GRE had potent radical-scavenging activities and neuroprotective effects in a rat model of stroke when it was pre- and co-administered. The optimal dosage may be around 300 mg/kg for oral administration.

## Introduction

The brain is the most susceptible organ to reactive oxygen species (ROS), one of the key pathological mechanisms in brain diseases, especially in ischemic brain stroke (Margaill et al. 2005). ROS are known to be produced in response to infiltrating leukocytes, glutamate toxicity, enzymes, and reperfusion (Lipton 1999). Reducing radical formation or removing radicals has protective effects in various brain ischemia models. To date, anti-oxidants have been investigated by many researchers for the development of stroke agents, and many of them have been developed from in vitro and in vivo experiments (Margaill et al. 2005). Furthermore, Edaravone®, which was used to treat acute stroke patients in Japan, was also a potent radical scavenger (Mizuno et al. 1998). Therefore, radical scavengers can probably be developed as stroke agents, and anti-oxidants used in traditional medicine could be the candidates for stroke therapy. We previously investigated potent anti-oxidant herbs that could be a potential resource of natural neuroprotective agents. Galla Rhois was found to be the most effective herb in various scavenging assays and was selected as a candidate for further study.

Galla Rhois is the excrescence produced by parasitic aphids that form host-adapted races or biotypes (Ren et al. 2008). Most aphids form gallnuts, which are abnormal outgrowths of plant tissue. Gallnuts provide habitat and food sources for aphids. Furthermore, some of them are also used as a medicine in traditional medicine in China and Korea (Ren et al. 2008). Galla Rhois are formed by aphids, primarily *Schlechtendalia chinensis* Bell (Homoptera: Aphididae), on the leaf of sumac, *Rhus javanica* L. (Sapindales: Anacardiaceae) (Lee et al. 1997). It has been used for treating diarrhea, seminal emission, excessive sweating, bleeding, and chronic cough in traditional Korean medicine (Shim et al. 2003; Kim et al. 2005; Song et al. 2002). It has been reported to have inhibitory effects on alpha-glucosidase (Shim et al. 2003); moreover, it has also been reported to have anti-anaphylactic (Kim et al. 2005), anti-thrombotic (Song et al. 2002), anti-viral (Nakano et al. 1998), anti-bacterial (Choi et al. 2005), and anti-plague effects (Cho et al. 2005), as well as radical-scavenging effects (Cha and Lee 1998).

The purpose of the current study was to investigate the neuroprotective effects and related mechanisms of Galla Rhois on ischemic brain disease. We confirmed the ROS-scavenging effects of Galla Rhois methanol extract (GRE) and applied it to transient middle cerebral artery occlusion (MCAo) rat models to investigate the protective effects, therapeutic time window, brain water content, and ROS-related mechanism.

## Materials and Methods

### Samples preparation

Galla Rhois was purchased from Omniherb Inc. (Deagu, Korea). Sample specimens of the material (WHR016) and extract (WHE016) are deposited at the Department of Herbal Pharmacology in Kyung Hee University, Seoul, South Korea.

Dried Galla Rhois (200 g) was added to 3000 mL of 85% MeOH, extracted under sonication for 120 min and filtered. After filtering, the remaining was added to 2000 mL of 85% MeOH, extracted once more for 90 min and filtered. Two filtered extracts were mixed together and evaporated in a vacuum at 60 °C and lyophilized with a freeze drier to obtain GRE (68.6 g). Distilled water was used both in the preparation of the solution for the in vitro and the in vivo tests.

### Radical-scavenging assay

The radical-scavenging activity of samples against 2,2-diphenyl-1-picrylhydrazyl (DPPH) (Sigma-Aldrich, www.sigmaaldrich.com) free radical was measured using methods reported previously (Chu et al. 2000) with minor changes. A MeOH solution of DPPH (1 mg DPPH in 14.3 mL MeOH) was mixed with 100.0 µL of vehicle or sample solution (1, 10, 100, and 1000 µg/mL). Optical density was measured at 517 nm after 30 min of incubation.

Superoxide radical was generated in a xanthine/xanthine oxidase system and measured by the nitroblue tetrazolium reduction assay (Gaulejac et al. 1999). The solutions of nitroblue tetrazolium (600 µM) (Sigma-Aldrich), xanthine (100 mM) (Sigma-Aldrich), and xanthine oxidase (0.1 unit/mL (Sigma-Aldrich) were prepared separately in a 0.1 M sodium phosphate buffer (pH 7.4). The vehicle or sample solution (1, 10, 100, or 1000 µg/mL) was added to the reaction solution, and the opitical density was measured at 570 nm after 10 min of incubation.

Nitric oxide radical scavenging assay was performed using Sodium nitroprusside (Sigma-Aldrich) as the NO donor. sodium nitroprusside, 5 mM in phosphate buffer saline was mixed with different concentrations of samples (1, 10, 100, and 1000 µg/mL) and incubated at 25 ^°^C for 30 min. After incubation, the solution was added to same volume of Griess reagent (1% sulphanilamide, 2% phosphoric acid, and 0.1% N-1-naphthylethelene diamine). The opitical density was measured at 546 nm.

Lipid-peroxidation (LPO) assay (thiobarbituric acid-reactive substances (TBARS) assay) was performed using brain homogenates. The homoginate was prepared by isolating rat brain (Male Sprague Dawley rat, 305 g), homogenizing with ice-cold 50 mM Tris-HCl buffer (pH 7.4) and setting the concentration to 0.2 mg/mL. 100 µL of different extract concentrations (1, 10, 100, and 1000 µg/ml) were added to 500 µL of the brain homogenate. LPO was initiated by adding 30 µL of 50 mM FeSO4 solution to the mixture. The reaction mixture was incubated at 37 ^°^C for 30 min. After incubation, 100 µL of trichloroacetic acid (Sigma-Aldrich) was added, and the mixture was centrifuged at 13,000 × *g* for five min. Thiobarbituric acid (500 µL) was added to 500 µL of the supernatant and incubated at 96 ^°^C for 10 min. The intensity of the resulting pink-colored complex was measured at 540 nm. TBARS values were calculated using standard 1,1,3,3-tetraethoxypropane (Sigma-Aldrich) and expressed as µmol/g tissue. All data of the assays were calculated by percent inhibition of the sample (opitical density or TBARS value) compared with the vehicle-treated (opitical density or TBARS value).

### Animals

All surgical procedures were conducted according to the animal welfare guidelines of the US National Institutes of Health and the Korean Academy of Medical Sciences. This study was approved (KHUASP (SE)-10-027) by the Kyung Hee University Institutional Animal Care and Use Committee. Rats were housed under controlled temperatures (22 ± 2 ^°^C), constant humidity, 12:12 L:D photoperiod, and food and water were available ad libitum. Food was withheld from male Sprague Dawley rats overnight, but they had free access to water before surgery.

### Surgical procedure

Male Sprague Dawley rats (290 ± 10 g) were anesthetized with chloral hydrate (400 mg/kg, i.p.). Rectal temperature was controlled at 37.0 ± 0.5 ^°^C throughout the experiment with a heating lamp. The transient focal cerebral ischemia rat model was produced by a modified intraluminal suture method (Longa et al. 1989). Briefly, a silicone coated 4–0 nylon monofilament (0.36 µm in diameter) was introduced into the external carotid artery at least 18–19 mm from carotid bifurcation. After 2 hours of MCAo, the suture was withdrawn to allow reperfusion for 22 hours.

### Grouping and sample treatment

Grouping and numbers of rats were as follows. Rats were divided into 4 groups (n = 8 each, vehicle-treated group, GRE 100, GRE 300, and GRE 1000 mg/kg treated group) to investigate the effects on brain damage; 8 groups (n = 8 each, 4 vehicle-treated groups [treated at -2 hours and 0 hours (pre-), at 0 hours and 2 hours (co-), at 3 hours and 5 hours (post-3 hours), and 6 hours and 8 hours (post-6 hours) after ischemia induction] and 4 GRE 300 mg/kg-treated groups at each treatment time point to investigate the therapeutic time range; 3 groups (n = 7, sham-operated group, vehicle-treated and GRE 300 mg/kg-treated group) to investigate the effects on sensory motor function and LPO, and 2 groups (n = 7, vehicle-treated and GRE 300 mg/kg-treated group) to investigate the effects on brain water content.

GRE was dissolved in distilled water and the decided doses and volume (100, 300, and 1000 mg/kg, 3 mL/kg) were orally administered twice at the 2-hour intervals and time points (pre-, co-, and post-treatment). The vehicle-treated group was administered distilled water by the same volume and at the same time as the GRE-treated groups. The sham-operated group was treated by the same method with MCAo induction except for insertion length (10 mm). To investigate the effects of GRE on brain water content, sensory motor function, and LPO, the effective dose of GRE (300 mg/kg) was used at 0 and 2 hours after ischemia induction.

### Measurement of infarct volume

Rats were sacrificed at 24 hours after occlusion with an overdose of chloral hydrate. Brain isolation, 2% 2,3,5-triphenyltetrazolium chloride (Sigma-Aldrich) staining and infarct volume measurement by Optimas 5.5 graphic analyzer (Media Cybernetics Inc., www.mediacy.com) were done by the method previously described by Bu et al. (2010). Infarct volume (%) was calculated by dividing the correlated infarct volume (mm3) by the total volume (mm3) of the ipsilateral hemisphere.

### Beam balance test

Beam balance test was performed at 22 hours after MCAo (Bu et al. 2010). Briefly, rats were laid on the middle of a wooden square bar (2.5 cm wide, 122 cm long, 42 cm high) for 30 sec and scored as follows: rat is unable to stay on the beam = 0 points; rat is unable to move, but able to stay on the beam = 1 point; rat tries to turn to left or right side of the beam = 2 points; rat turns to left or right side and walks on the beam with more than 50% foot-slips of the affected hindlimb = 3 points; rat traverses the beam with more than one foot-slip, but less than 50% = 4 points; rat has only one slip of the hind limb = 5 points; rat traverses the beam without any slips of the hind limb = 6 points.

### Measurement of LPO

Ipsilateral hemispheres were measured as wet weight after isolation. The degree of LPO of ipsilateral hemisphere was assayed by the same in vitro TBARS assay method, except for sample treatment and FeSO4 treatment into solution. TBARS values were calculated using standard 1,1,3,3-tetraethoxypropane and expressed as µmol/g tissue.

### Measurement of brain water content

Brains were isolated quickly 24 hours after MCAo, the pons and olfactory bulb (bregma -2 to +10) were isolated, and the brains were divided into both ipsilateral and contralateral hemispheres. The wet weight of each hemisphere was measured on a chemical balance (Ohaus Corporation, us.ohaus.com) within 2 min after isolation. All hemispheres were dried at 105 ^°^C in a dry chamber for 48 hours and then measured as dry weight. The water content of each hemisphere was calculated as a percentage (%) by dividing the difference between the wet and dry weight by the wet weight.

**Figure 1.**
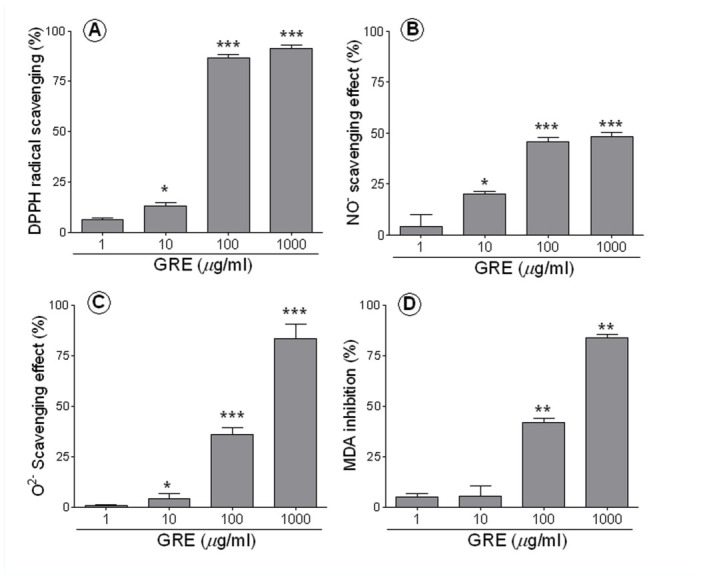
Inhibitory effects of GRE on radicals and LPO. DPPH radical scavenging effect was measured using a MeOH solution of DPPH (A), Superoxide radical scavenging effect was measured using the solutions of nitroblue tetrazolium (600 µM), xanthine (100 mM), and xanthine oxidase (0.1 unit/mL) in a 0.1 M sodium phosphate buffer (B). Nitric oxide radical was done using sodium nitroprusside in phosphate buffer saline (C) and LPO was done using rat brain homogenates, which were incubated TCA and TBA after adding FeSO4 (D). All assays measured opitical density by spectrophotometer. All values are percent inhibition compared to control and expressed by mean **±** SD. Asterisks (*) represent statistical differences from control: * *p* < 0.05, ** *p* < 0.01, and *** *p* < 0.001 when comparing with control by Student's unpaired 2-tailed *t*-test. High quality figures are available online.

### Statistical analysis

Results are expressed as mean ± SD and the graphs were expressed as box-whisker plots for expressing the mean and distribution of the data. The box indicates the middle 50% of the data and the whisker indicates the lowest and highest data point. Statistical analysis was done using GraphPad Prism 4.0 (GraphPad Software, www.graphpad.com). Comparison between groups was done by means of one-way ANOVA using Tukey's post hoc test or Dunnett's test, and comparison between two groups was by Student's unpaired 2-tailed *t-*test. Values of *p* < 0.05 were considered statistically significant.

## Results

### Radical scavenging effects of GRE

GRE had a dose-dependent effect on DPPH, superoxide, nitric oxide, and LPO: 1, 10, 100, and 1000 µg/mL of GRE showed 6.2, 13.3, 86.7, and 91.2% efficacy, respectively, against DPPH radical (*p* < 0.05 in 100 mg/kg, *p* < 0.001 in 1000 µg/mL vs. vehicle-treated, Figure 1A); 0.8, 4.2, 36.2, and 83% efficacy, respectively, against superoxide radical (*p* < 0.05 in 100 µg/mL, *p* < 0.001 in 1000 µg/mL vs. vehicle-treated, Figure 1B); and 0, 19.2, 44.1, and 46.4% efficacy, respectively, against nitric oxide radical (*p* < 0.05 in 100 µg/ml, *p* < 0.001 in 1000 µg/mL vs. vehicle-treated, Figure 1C). The 100 and 1000 µg/mL GRE-treated groups showed 33.1% and 83% inhibition (20.2 ± 0.8 and 5 ± 0.4 µmol/g tissue, respectively), while the vehicle-treated group showed 30.2 ± 1.4 µmol/g tissue of TBARS (*p* < 0.001 in 100 and 1000 µg/ml vs. vehicle-treated, Figure 1D). However, the effect on nitric oxide was lower than the effect on other radicals.

**Figure 2.**
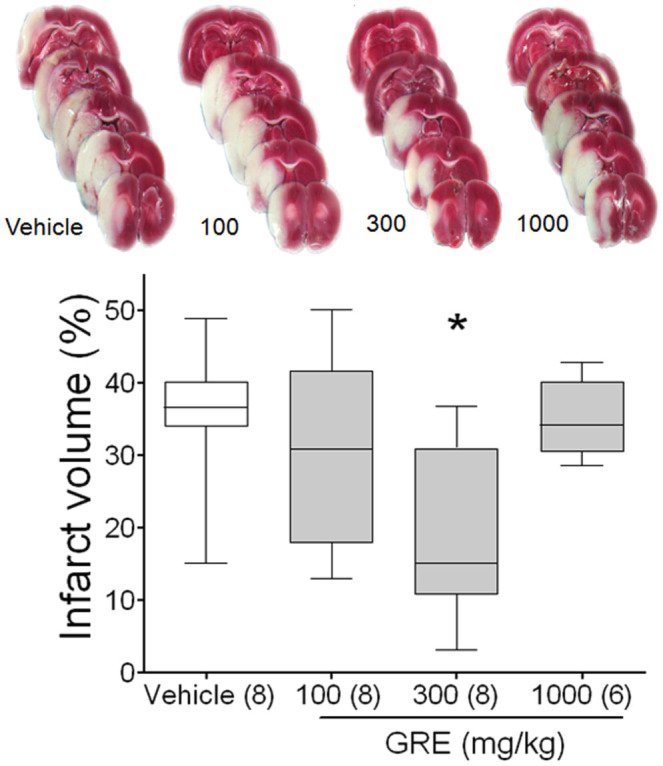
Protective effects of GRE on MCAo rat model. Photographs are representative coronal brain sections stained with 2,3,5-triphenyltetrazolium chloride after two hours of MCAo and 24 hours of reperfusion showing infarct area (white) and intact area (red). Graph shows the neuroprotective effects of GRE on MCAo in rats. MCAo was induced by intra-luminal suture method (two hours of ischemia and 22 hours of reperfusion) and samples were treated at 0 and two hours after MCAo. Brain sections were made by isolating at 22 hours after MCAo and stained by 2% 2,3,5-triphenyltetrazolium chloride. The infarct volume was calculated by dividing the correlated infarct volume (mm3) by the total volume (mm3) of the ipsilateral hemisphere. The data are expressed as box-whisker plots: the box represents the 25–75th percentiles and the bar across the box indicates the average of each group. The whiskers on each box represent the lowest and highest data. * *p* < 0.05 when comparing with vehicle treated group in one-way ANOVA with Tukey's test. High quality figures are available online.

### Neuroprotective effects of GRE on the MCAo rat model

Coronal sections were obtained by cutting the brain at a distance of 4, 6, 8, 10, 12, and 14 mm from the rostral extremity of the frontal cortex. The white areas, representing the infarction regions in these sections, generally extended from the caudoputamen, parietal cortex, and temporal cortex to the penumbral legion in MCAo. GRE 300 mg/kg-treated rats clearly showed less brain infarction than vehicle-treated rats (Figure 2).

The infarct volume of the vehicle-treated group was 35.6 ± 3.66%. Oral administration of GRE 100, 300, and 1000 mg/kg reduced infarct volume to 30.5 ± 5.12, 19.14 ± 4.59, and 34.9 ± 2.2%, respectively (*p* < 0.05 in the GRE 300 mg/kg-treated group, Figure 2). However, the 1000 mg/kg treatment was ineffective in this procedure. GRE 300 mg/kg showed 37.5% neuroprotection compared with vehicle-treated group (Figure 2). Two rats in the GRE 1000 mg/kg-treated group died during the reperfusion period due to hemorrhage and severe damage.

### Effect of GRE on behavior

**Figure 3.**
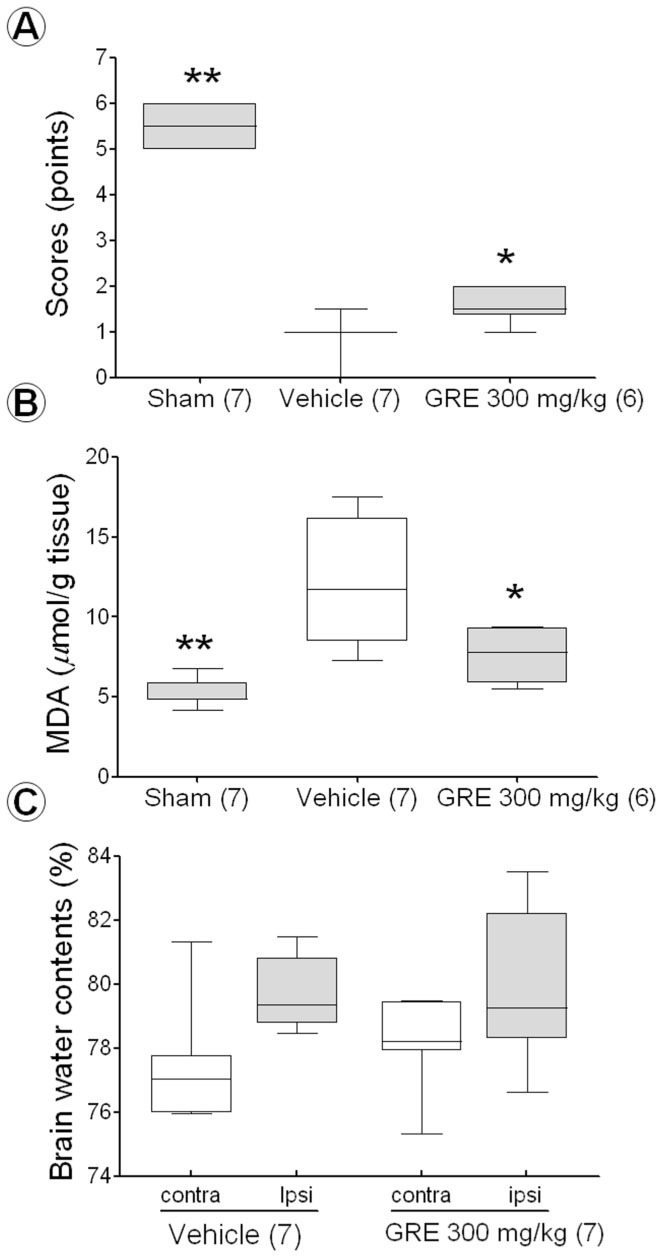
The effect of GRE 300 mg/kg on the Beam balance beam test (A), lipid-peroxidation (B), and brain water contents (C) after MCAo. Balance beam test was performed at 22 hours after MCAo. The behavior status was scored by 6 grades (A). LPO and brain water content was measured at 24 hours after MCAo. LPO was measured by TBARs assay using TBA and TCA (B). Brain water content was calculated as a percentage (%) by dividing the difference between the wet and dry weight by the wet weight (C). The data are expressed as box-whisker plots: the box represents the 25–75th percentiles, the bar across the box indicates the average of each group, and the whiskers on each box represent the lowest and highest data. Contra and ipsi indicate contralateral and ipsilateral hemisphere, respectively. The numbers in parentheses are the numbers of rats. * *p* < 0.05 and ** *p* < 0.01 when compared with the vehicle-treated group in one-way ANOVA with Dunnett's test. High quality figures are available online.

Rats in the vehicle-treated group scored significantly lower on the balance beam test than rats in the sham-operated group (1.1 ± 0.17 points vs. 5.5 ± 0.25 points; *p* < 0.01); however, rats that received GRE 300 mg/kg scored higher than those in the vehicle-treated group (1.6 ± 0.10 points and 1.1 ± 0.17 points; *p* < 0.01, Figure 3A). One rat in the vehicle and GRE-treated group died due to perforation by suture.

### Effect of GRE on LPO after MCAo

The TBARS level of the vehicle-treated group was increased 11.8 ± 1.58 µmol/g tissue compared with 5.2 ± 0.35 µmol/g tissue of sham-operated group (*p* < 0.01). GRE 300 mg/kg treatment reduced to 7.63 ± 0.77 µmol/g tissue compared with vehicle-treated group (*p* < 0.05, Figure 3B).

### Effect of GRE on brain water content after MCAo

Two hours of MCAo followed by 22 hours of reperfusion increased the brain water content of the ipsilateral hemisphere compared to the contralateral hemisphere. The mean water content of the contralateral and ipsilateral hemispheres was 77.3 ± 0.79% and 79.8 ± 0.47%, respectively, in the vehicle-treated group, and 78.2 ± 0.59% and 80.1 ± 0.99% in the GRE 300 mg/kg-treated group. The GRE 300 mg/kg treatment did not have decreased brain water content (Figure 3C).

### Effect of GRE according to treatment time

**Figure 4.**
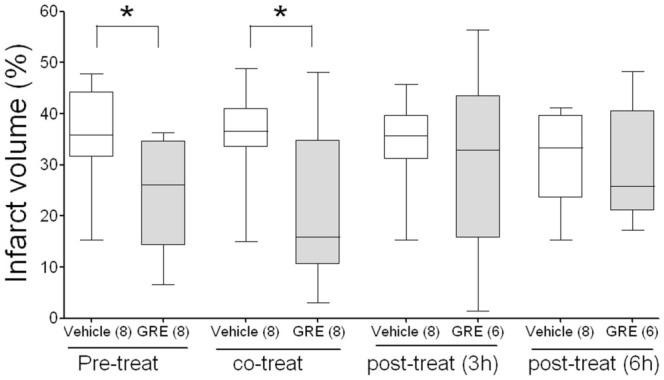
The effects of GRE 300 mg/kg on brain infarction after MCAo by the treatment time. GRE and vehicles were orally administered at -2 hours and 0 hours (pretreatment), at 0 hours and 2 hours (cotreatment), at 3 hours and 5 hours (post-treatment (3 hours)) and at 6 hours and 8 hours (post-treatment (six hours) after MCAo induction. Brain sections were made by isolating at 22 hours after MCAo and stained by 2% 2,3,5-triphenyltetrazolium chloride. The infarct volume was calculated by dividing the correlated infarct volume (mm3) by the total volume (mm3) of the ipsilateral hemisphere. The data are expressed as box-whisker plots: the box represents the 25–75th percentiles and the bar across the box indicates the average of each group. The whiskers on each box represent the lowest and highest data. The numbers in parentheses are the numbers of rats. * *p* < 0.05 when compared with each vehicle-treated group in one-way ANOVA with Dunnett's test. High quality figures are available online.

The vehicle-treated groups at pre-, co-, post-3-hour, and post-6-hour were 35.6 ± 3.84%, 35.6 ± 3.67%, 34.2 ± 3.43%, and 31.8 ± 3.47%, respectively, while the GRE-treated groups at same time points were 23.9 ± 4.09%, 22.4 ± 5.28%, 30.6 ± 8.29%, and 29.6 ± 5.16%, respectively. The pre-treatment and co-treatment groups had similar effects on MCAo (*p* < 0.05); however, the post-3-hour and post-6-hour treatment groups did not show significance (Figure 4). One rat in each GRE post-treated group died; one of perforation and the other of unknown cause.

## Discussion

In the current study, GRE showed potent scavenging effects on DPPH, superoxide radicals, and nitric oxide radicals and an inhibitory effect on LPO in rat brain homogenate. In cerebral ischemia, it reduced brain infarct volume when used before and during occlusion and improved behavioral function by inhibition of LPO. However, it did not reduce brain infarct when used at 3 or 6 hours after occlusion and did not reduce brain edema even when used during occlusion.

DPPH scavenging effect is defined as the ability to donate a hydrogen atom. In the current study, GRE was found to be more effective than ascorbic acid (49.3% and 82.5%, 100 and 1,000 µg/mL of ascorbic acid, data not shown), which is in agreement with a previous report (Cha and Lee 1998). However, the system of the DPPH radical assay is different from an in vivo system, such as pH and solutions (Sanchez-Moreno 2002) and other radical systems that have not been adapted to GRE. Thus, the effects of GRE on other radicals or other systems need to be investigated. Superoxide itself is a relatively weak oxidant. However, it induces the formation of other stronger reactive radicals, such as hydroxyl radical and single oxygen species. Superoxide scavenging effect is the inhibition of cell damage progression by the radicals involved (Chen et al. 2001). Nitric oxide radical scavenging effect is the inhibitory effect on the inflammatory process and on the formation of peroxinitrate, which induces DNA fragmentation, cell damage, and neuronal cell death in ischemic and certain degenerative brain diseases (Moncada et al. 1991). Inhibitory effect on LPO is the inhibitory effect on the toxicity of radicals on the cell membrane, and malondialdehyde is one of the major products of LPO. TBARS assay is the most common method for assessing amounts of malondialdehyde in biological samples (Janero 1990). From the results of the radical scavenging assay, it seems that GRE has potent radical scavenging effects and could be applied to the in vivo rat model of stroke.

Oral administration of 300 mg/kg of GRE twice at 0 and 2 hours after MCAo reduced the brain infarct volume by 46.2% compared with the vehicle-treated group, but treatment with GRE at 1000 mg/kg did not have any such effect. The advantages of the MCAo rat model used in the current study are that small animals can be used, experiments can be performed under controlled conditions, and the pathophysiology of stroke patients can be mimicked (Longa et al. 1989). There are two types of cell death in MCAo rats. Cell death in the ischemic core is known as necrotic cell death, and that in the penumbra is known as apoptotic cell death (Longa et al. 1989). In the ischemic core, the only way to recovery is re-canalization within 3 hours. It is almost impossible to recover after 3 hours of ischemia (Lipton 1999). On the other hand, it is possible to recover from apoptotic cell death in the penumbra after 3 hours of ischemia. Thus, many researchers have focused on protection from apoptotic cell death. Although apoptotic cell death is caused by a variety of biochemical mechanisms, this study only focused on oxidative stress (Margaill et al. 2005).

Although GRE was not fractionated, the major anti-oxidative compounds—gallic acid methyl ester, protocatechuic acid, and 1,2,3,4,6-penta-O-galloyl-b-D-glucose—might be the major compounds responsible for the neuroprotective effect (Cha et al. 2000). In addition, the effects on the inflammatory reaction in cerebral ischemia must be investigated because oxidative stress also results from perturbed mitochondrial metabolism and inflammatory responses (Karageuzyan 2005).

In the current study, 1000 mg/kg GRE did not have any neuroprotective effect, possibly due to tannin toxicity, as Galla Rhois contains > 60% tannins. Tannins, which are water-soluble phenolic compounds, contain a large number of hydroxyl or other functional groups (Haslam 2007). Tannins of plant material have beneficial pharmacological effects on the one hand, but are toxic on the other hand. Low-dose intake of the correct kind of tannins may be good for human health due to their anti-oxidative, anti-inflammatory, anti-bacterial, hypotensive, and hypocholesterolemic effects (Chung et al. 1998). However, high-dose intake of tannins induces cancer formation, hepatotoxicity, alteration of excretion of certain cations, and increased excretion of proteins and essential amino acids; it is also harmful to the mucosal lining of the gastrointestinal tract (Haslam 2007). Therefore, it may be important to determine the correct dosages of tannins or tannin-containing agents for promoting optimal health conditions. Furthermore, the physiologic condition of rats after MCAo induction was very different compared to normal conditions because of operation-related brain damage. We previously learned that administration of 1000 mg/kg of GRE twice at 2-hour intervals for 3 days did not show toxic effects under normal conditions (no death in 8 rats, data not shown). In the current study, it showed not only ineffectiveness but also toxicity (2 of 8 rats dies; 25%). A human dose of 1000 mg/kg would mean that a 50-kg human would take 50 g of extract, which is a very high amount. Therefore, it could be suggested that the optimal and effective dosage in the MCAo rat model might be around 300 mg/kg; thus, we performed the subsequent study with 300 mg/kg GRE. Treatment with 300 mg/kg GRE improved sensory motor function compared with treatment with the vehicle. The balance beam test was used to determine the effect of agents on the functional deficits of rats subjected to neuronal damage (Puurunen et al. 2001). The effects of GRE on post-ischemic function suggest that it may improve behavioral deficits and reduce the damage in the area related to synaptic transmission including the cortex (Tamura et al. 1981). The current results may provide evidence for the neuroprotective effects of GRE on brain infarct.

In the current study, treatment with GRE 300 mg/kg did not show any inhibitory effects on brain edema, which is known to begin to increase as soon as three hours after MCAo and reach maximal levels at 48 hours after ischemia (Gotoh et al. 1985). Brain edema could increase mechanical cranial pressure and reduce blood flow to the damaged area, leading to secondary brain damage. Thus, reduction of brain edema not only means reduction of water influx to the brain but also provides indirect neuroprotection (Gerriets et al. 2009). The relationship between ROS and brain edema is somewhat well investigated (Jian et al. 2005). However, the mechanisms of brain edema are very complicated and are not yet fully investigated. To date, aquaporins, matrix metalloproteinases, integrins, and vascular endothelial growth factors could be the main candidate mechanisms in addition to ROS (Nag et al. 2009). The ineffective nature of GRE on brain edema might be due to mechanisms other than ROS, and it could also be suggested that the effect of GRE on brain infarct came from effects on the direct mechanisms of brain damage and not by indirect reduction of brain edema.

The mechanism study using the TBARS assay revealed that treatment with GRE 300 mg/kg reduced LPO in ischemia and reperfusion. In the case of transient focal cerebral ischemia, ROS-induced LPO increased two hours after reperfusion and was highest at 24 hours after reperfusion (Sinha et al. 2002). The current result is consistent with the neurological and histological results. Thus, it could be suggested that the LPO inhibitory effect of GRE is a possible mechanism in the neuroprotective effects in cerebral ischemia.

In the therapeutic window study, GRE showed neuroprotective effects in pre-treatment and co-treatment; however, it did not have an effect in post-treatment at three hours (one hour after reperfusion) or six hours after ischemia (four hours after reperfusion). The effect seen with pre- and co-treatment means that GRE has preventive and therapeutic effects in MCAo. One of the keys in the treatment of transient focal cerebral ischemia by anti-oxidants is therapeutic time window, since the occurrence of oxidative stress is closely related to the onset of ischemia and reperfusion. During ischemia, a two-fold increase in radicals occurs within 15 min of brain ischemia onset (Sinha et al. 2002) and persisted throughout 2–3 hours of ischemia. Reperfusion after 1 or 2 hours induced a burst of radical formation that lasted 40 min and persisted for the remaining two hours (Fabian et al. 1995). Radical scavenger N-acetyl cysteine was more effective in pre-treatment than co-treatment with reperfusion in transient focal cerebral ischemia (Carroll et al. 1998). Putting together these studies and our results, it could be suggested that radical scavengers might show neuroprotective effects on the transient focal cerebral ischemia rat model, but must be orally administered before, simultaneously, or possibly within four hours of occlusion, and that GRE treatment in the stroke rat model has to be done simultaneously with MCAo.

In conclusion, GRE, which showed radical scavenging effects, protected against ischemia-induced brain damage and sensory-motor functional by inhibitory effects on LPO. The optimal dosage was 300 mg/kg and the therapeutic time is between 0 hours and three hours after MCAo in the case of oral administration. The current results suggested that Galla Rhois could be a candidate natural resource for stroke drug developments.

## Abbreviations

**GRE**,: Galla Rhois 85% methanol extract;
**ROS**,: reactive oxygen species;
**DPPH**,: 2,2-diphenyl-1-picrylhydrazyl;
**LPO**,: lipid-peroxidation;
**MCAo**,: middle cerebral artery occlusion;
**TBARS**,: thiobarbituric acid-reactive substances
